# Association between triglyceride glucose index and breast cancer in 142,184 Chinese adults: findings from the REACTION study

**DOI:** 10.3389/fendo.2024.1321622

**Published:** 2024-06-06

**Authors:** Xueyan Wu, Shuangyuan Wang, Lin Lin, Xiaojing Jia, Chunyan Hu, Hongyan Qi, Hong Lin, Ruizhi Zheng, Mian Li, Yu Xu, Min Xu, Lulu Chen, Tianshu Zeng, Ruying Hu, Zhen Ye, Lixin Shi, Qing Su, Xuefeng Yu, Li Yan, Tiange Wang, Zhiyun Zhao, Jie Zheng, Guijun Qin, Qin Wan, Gang Chen, Meng Dai, Xulei Tang, Zhengnan Gao, Feixia Shen, Xuejiang Gu, Zuojie Luo, Yingfen Qin, Li Chen, Xinguo Hou, Yanan Huo, Qiang Li, Guixia Wang, Yinfei Zhang, Chao Liu, Youmin Wang, Shengli Wu, Tao Yang, Huacong Deng, Jiajun Zhao, Yiming Mu, Guang Ning, Weiqing Wang, Yufang Bi, Yuhong Chen, Jieli Lu

**Affiliations:** ^1^ Department of Endocrine and Metabolic Diseases, Shanghai Institute of Endocrine and Metabolic Diseases, Ruijin Hospital, Shanghai Jiao Tong University School of Medicine, Shanghai, China; ^2^ Shanghai National Clinical Research Center for Endocrine and Metabolic Diseases, Key Laboratory for Endocrine and Metabolic Diseases of the National Health Commission of the PR China, Shanghai National Center for Translational Medicine, Ruijin Hospital, Shanghai Jiao Tong University School of Medicine, Shanghai, China; ^3^ Union Hospital, Tongji Medical College, Huazhong University of Science and Technology, Wuhan, China; ^4^ Institute of Chronic Diseases, Zhejiang Provincial Center for Disease Control and Prevention, Hangzhou, China; ^5^ Department of Endocrinology, Affiliated Hospital of Guiyang Medical College, Guiyang, China; ^6^ Xinhua Hospital Affiliated to Shanghai Jiaotong University School of Medicine, Shanghai, China; ^7^ Tongji Hospital, Tongji Medical College, Huazhong University of Science and Technology, Wuhan, China; ^8^ Sun Yat-sen Memorial Hospital, Sun Yat-sen University, Guangzhou, China; ^9^ Department of Endocrinology, The First Affiliated Hospital of Zhengzhou University, Zhengzhou, China; ^10^ Department of Endocrinology, The Affiliated Hospital of Southwest Medical University, Luzhou, China; ^11^ Fujian Provincial Hospital, Fujian Medical University, Fuzhou, China; ^12^ Department of Endocrinology, The First Hospital of Lanzhou University, Lanzhou, China; ^13^ Department of Endocrinology, Dalian Municipal Central Hospital, Dalian, China; ^14^ Department of Endocrinology, The First Affiliated Hospital of Wenzhou Medical University, Wenzhou, China; ^15^ Department of Endocrinology, The First Affiliated Hospital of Guangxi Medical University, Nanning, China; ^16^ Department of Endocrinology, Qilu Hospital of Shandong University, Jinan, China; ^17^ Department of Endocrinology, Jiangxi Provincial People's Hospital Affiliated to Nanchang University, Nanchang, China; ^18^ Department of Endocrinology, The Second Affiliated Hospital of Harbin Medical University, Harbin, China; ^19^ Department of Endocrinology, The First Hospital of Jilin University, Changchun, China; ^20^ Department of Endocrinology, Central Hospital of Shanghai Jiading District, Shanghai, China; ^21^ Department of Endocrinology, Jiangsu Province Hospital on Integration of Chinese and Western Medicine, Nanjing, China; ^22^ Department of Endocrinology, The First Affiliated Hospital of Anhui Medical University, Hefei, China; ^23^ Department of Endocrinology, Karamay Municipal People's Hospital, Xinjiang, China; ^24^ Department of Endocrinology, The First Affiliated Hospital of Nanjing Medical University, Nanjing, China; ^25^ Department of Endocrinology, The First Affiliated Hospital of Chongqing Medical University, Chongqing, China; ^26^ Department of Endocrinology, Shandong Provincial Hospital Affiliated to Shandong University, Jinan, China; ^27^ Department of Endocrinology, Chinese People’s Liberation Army General Hospital, Beijing, China

**Keywords:** insulin resistance, triglyceride, glucose, breast cancer, Chinese population

## Abstract

**Background:**

The triglyceride glucose (TyG) index has been associated with an increased risk in breast cancer. However, this association remains unclear among the Chinese population. This study aimed to investigate whether the TyG index is associated with the risk of prevalent breast cancer in Chinese women.

**Methods:**

This cross-sectional study included 142,184 women from the REACTION (Risk Evaluation of Cancers in Chinese Diabetic Individuals: A Longitudinal) Study, which recruited adults aged 40 years or older from 25 centers across mainland China between 2011 and 2012. The TyG index was calculated according to the formula: Ln (fasting triglycerides [mg/dL] × fasting glucose [mg/dL]/2). Multivariable-adjusted logistic regression models were used to evaluate odds ratios (ORs) and 95% confidence intervals (CIs) regarding the associations between the TyG index and breast cancer.

**Results:**

Multivariable-adjusted logistic regression analysis showed that compared with the lowest quartile of the TyG index, the highest quartile of the TyG index was significantly associated with an increased risk of prevalent breast cancer, with an OR (95% CI) of 1.61 (1.19–2.17). In the stratified analysis, the association of each 1 SD increase in the TyG index with risk of prevalent breast cancer was more dominant in individuals with menarche at age 13–17, those who were postmenopausal, those with a history of breastfeeding, and those who had two to four children, with the ORs (95% CIs) of 1.35 (1.09–1.68), 1.27 (1.05–1.54), 1.26 (1.05–1.52), and 1.32 (1.08–1.62), respectively. Moreover, among those without discernible insulin resistance (homeostatic model assessment-insulin resistance [HOMA-IR] ≥2.5), hyperglycemia and dyslipidemia, each 1 SD increase in the TyG index was associated with a 1.36-fold increase in breast cancer risk, with an OR (95% CI) of 2.36 (1.44–3.87).

**Conclusion:**

The TyG index is significantly associated with the prevalent breast cancer risk among middle-aged and elderly Chinese women.

## 1 Introduction

Breast cancer is the most popular cancer among women worldwide, with an estimated 2.3 million new cases (11.7%) of all the estimated 19.3 million new cancer cases in 2020, according to the latest 2020 global cancer statistics from the International Agency for Research on Cancer (IARC) (1). In China, female breast cancer claimed the unfortunate distinction of being the most common cancer, contributing to 16.7% of all newly diagnosed cancer cases (2). Identifying factors or related indices associated with the development of breast cancer is of great societal impact.

Insulin resistance (IR) plays a key role in the pathophysiology of breast cancer (3). IR, which leads to glucose intolerance, elevated homeostatic model assessment-insulin resistance (HOMA-IR), and compensatory hyperinsulinemia, is thought to be a central cause of obesity-related cancer onset and associated with unfavorable prognosis in postmenopausal women with breast cancer (4, 5). Previous studies have investigated the association between breast cancer risk and IR markers; however, these studies mostly relied on glucose, insulin, C-peptide, or HOMA-IR as related markers, and the association remains inconsistent (5–7). The triglyceride glucose (TyG) index is a reliable biochemical marker to assess IR, which has inexpensive and easy-to-use properties, compared with traditional hyperinsulinemic-euglycemic clamp (HIEC) (8). Currently, the population-based studies on the association of the TyG index with breast cancer risk are limited. A recent study has reported a non-linear dose–response relationship between TyG index and breast cancer among Indonesians (9). However, no significant association was observed in the European population (10). In addition, there is a lack of evidence in the Chinese population, and the relationship between TyG index and the risk of breast cancer is not yet clear.

Therefore, our study set out to explore the association between TyG index and breast cancer in a cross-sectional study of the general Chinese population. Additionally, we aimed to determine whether the TyG index maintains its association with breast cancer risk in a population without IR as defined by the HOMA-IR.

## 2 Materials and methods

### 2.1 Study population

**Figure 1:**
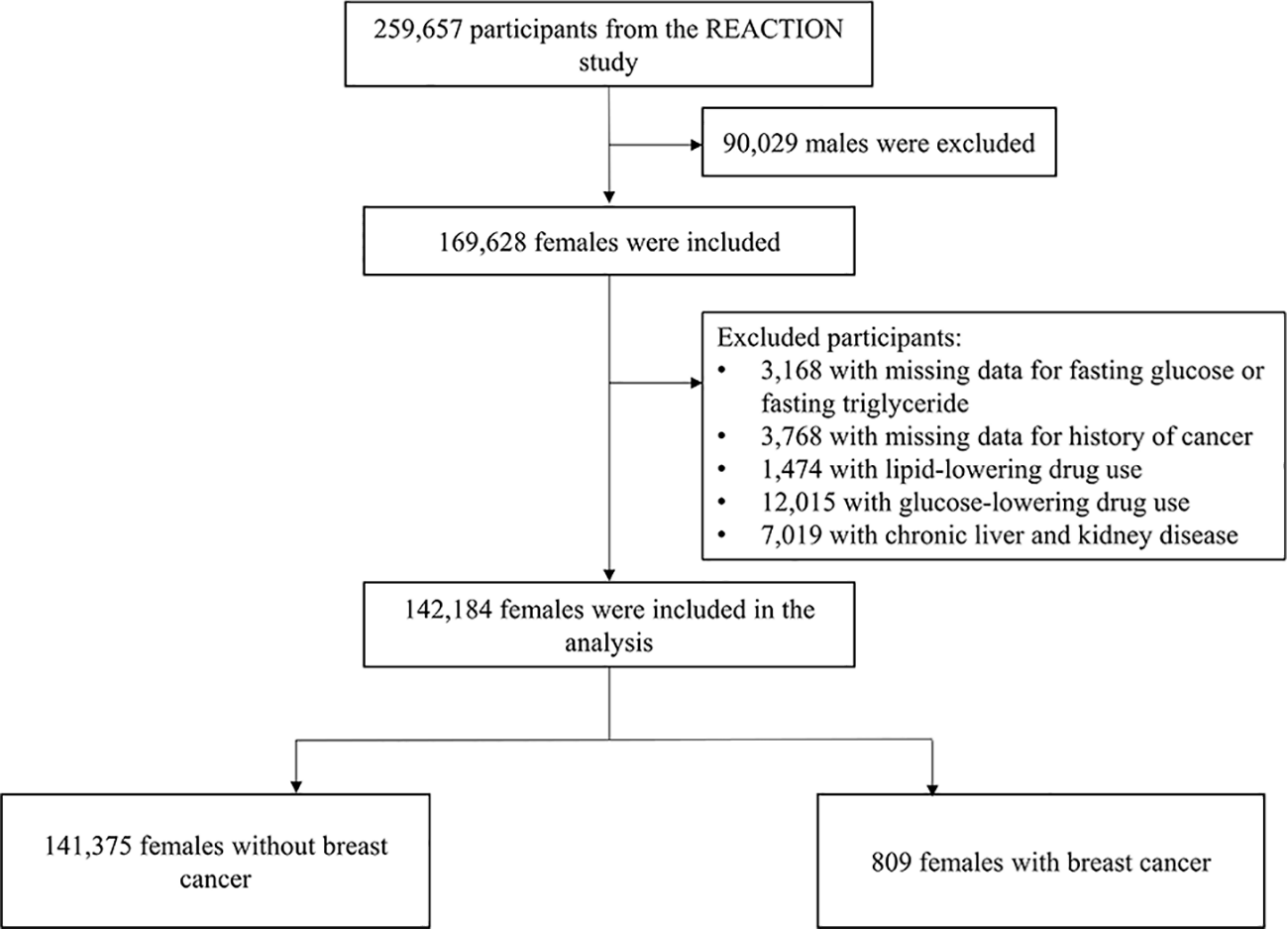
Participant flow diagram of this study.

The REACTION study was a population-based multicenter study, in which a total of 259,657 Chinese adults (aged ≥40 years) from 25 communities across mainland China were recruited to participate in the baseline survey during 2011 to 2012. The detailed design and methods of the study cohort have been described previously (11–13). Briefly, eligible participants were identified from the local residence registration records and was approached by trained community workers using a door-to-door invitation in the baseline survey during 2011 to 2012. There were no gender or race restrictions during the recruitment period, which ended up with 169,628 women. The current analysis was a cross-sectional design, provided only baseline data from the REACTION study baseline survey, and included only women. After excluding those who had missing data for fasting glucose or fasting triglyceride (n = 3,168), had missing data for history of cancer (n = 3,768), use lipid-lowering drugs (n = 1,474) and glucose-lowering drugs (n = 12,015), or had chronic liver and kidney diseases (n = 7,019), a total of 142,184 women were included in the final analysis (**Figure 1**).

The study conformed to the Institutional Review Board of the Ruijin Hospital, Shanghai Jiao Tong University School of Medicine. All participants provided written informed consent.

### 2.2 Data collection

The details of the data collection have been described previously (11). Trained investigators at each study center collected sociodemographic characteristics, lifestyle factors, and medical and family histories from a standardized questionnaire through personal interviews. Anthropometric measurements such as weight and height were performed by trained nurses according to a standard protocol. Women who had smoked one cigarette per day or seven per week regularly during the past 6 months were defined as current smokers, and those who had consumed alcohol once per week regularly during the past 6 months were defined as current drinkers. The type and frequency of smoking and alcohol consumption were recorded. Educational levels were divided into high school or above versus less than high school. The International Physical Activity Questionnaire was used to estimate intensity, duration, and frequency of physical activities by using the metabolic equivalent time per week. The validated dietary questionnaire was designed to collected participants’ dietary habits, frequency, and quantity information over the past 12 months, including red meat, fruits, vegetables, and dairy of typical food items. Women were asked about their reproductive history, including age at menarche, menopausal status, number of childbirths, and breastfeeding status.

Blood sampling was performed in the morning after an overnight fast of at least 10 h, and the samples were stored in dry ice at −80°C for transport to the central laboratory located at the Shanghai Institute of Endocrine and Metabolic Diseases that was certified by the College of American Pathologists. Glycated hemoglobin (HbA1c) was assayed by means of high-performance liquid chromatography method (Variant II and D-10 Systems; Bio-Rad, Hercules, CA). Fasting insulin (FINS) was measured with chemiluminescent immunoassay (i2000SR system, ARCHITECT ci16200 analyzer; Abbott Laboratories). Total cholesterol (TC), triglycerides (TG), high-density lipoprotein cholesterol (HDL-C), and low-density lipoprotein cholesterol (LDL-C) were measured on an autoanalyzer (c16000 system, ARCHITECT ci16200 analyzer; Abbott Laboratories, Lake Bluff, IL) in the central laboratory. In addition, all participants underwent an oral glucose tolerance test (OGTT). Plasma samples were obtained at 0 h and 2 h during the test, and blood glucose levels were assessed by glucose oxidase or hexokinase assay. HOMA-IR was calculated using the mathematical formula as follows: homeostatic model assessment of insulin resistance (HOMA-IR) = fasting plasma glucose (FPG) (mmol/L) × FINS (µU/mL)/22.5 (14).

### 2.3 Definitions and diagnostic criteria

The TyG index was calculated according to the formula that was previously published: TyG index = Ln [fasting TG (mg/dL) × FPG (mg/dL)/2] (10). Body mass index (BMI) was calculated as body weight in kilograms divided by body height squared in meters (kg/m^2^). Diagnosis of breast cancer was self-reported and further validated by reviewing medical records or pathology reports.

### 2.4 Statistical analysis

Data on the basic characteristics are presented as means ± standard deviations (SD) for normally distributed continuous variables or median (interquartile range) for continuous variables with a skewed distribution, and frequency (proportion) for categorical variables. Comparison of continuous variables was analyzed using one-way analysis of variance (ANOVA) and χ^2^ tests for categorical variables. Correlations between metabolic factors and TyG index were assessed using Spearman’s correlation analysis. Due to the cross-sectional nature of this study, there was a lack of follow-up time for breast cancer and Cox analysis could not be performed. Therefore, multivariable adjusted logistic regression analysis was used to evaluate the odds ratio (OR) and 95% confidence interval (CI) of TyG index and breast cancer. The multivariable model was adjusted for age, BMI, smoking status (current smoker or not), drinking status (current drinker or not), physical activity (moderate to vigorous or none to mild), family history of breast cancer, healthy diet (yes or no), 2-h plasma glucose (2h-PG), HbA1c and HDL-C in model 1 and further adjusted for age at menarche, menopausal status (yes or no), breastfeeding (yes or no), and number of childbirths in model 2. The TyG index entered the model either as a continuous variable with each 1 SD increase or as a categorical variable that was divided into quartiles, with the lowest quartile (Q1) as the reference group. Univariate logistic regression models were built to identify the associations between TyG index vs. TG, HbA1c, and FPG with breast cancer. *P* for trend across quartiles was calculated using ordinal values in separate models. Sensitivity analysis was conducted using Poisson regression with robust standard errors to compute prevalence ratios (PRs) and 95% CIs for associations between TyG index and breast cancer, adjusting for confounders.

Subgroup analysis was further performed to evaluate the potential effect of metabolic and reproductive factors on the association of TyG index each 1 SD increase and breast cancer. A likelihood ratio test was used to calculate the *P* value for interaction by comparing models with and without the interaction terms. In addition, several sensitivity analyses were conducted to test the role of TyG index on breast cancer in different metabolic states. We repeated the analyses excluding individuals with IR (HOMA-IR ≥2.5), hyperglycemia (FPG ≥6.1 mmol/L or 2h-PG ≥7.8 mmol/L or diagnosed with type 1 or type 2 diabetes or pre-diabetes), and dyslipidemia (TC ≥6.2 mmol/L or LDL-C ≥4.1 mmol/L or HDL-C <1.0 mmol/L or TG ≥2.3 mmol/L), respectively. In addition, to further explore whether this association is still significant in women with relatively healthy metabolic status, individuals with discernible IR, hyperglycemia, and dyslipidemia at the same time were also excluded. All statistical analyses were performed using SAS (version 9.4, SAS Institute Inc, Cary, NC). A two-tailed *P* < 0.05 was considered statistically significant.

We also used R (v 4.2.1) to construct restricted cubic spline (RCS) analysis using four knots to assess the non-linear dose–response relationship between TyG index and breast cancer after full adjustment.

## 3 Results

Among the 142,184 women included, the mean (SD) age of the study population was 56.37 ± 9.30 years old and 809 (0.57%) individuals had breast cancer. **Table 1** presents the baseline characteristics of the study population according to the quartile of the TyG index. On average, those with a lower TyG index were much younger and more educated, more likely to have ideal physical activity and healthy dietary habits, and tended to have lower levels of BMI, plasma glucose, TG, TC, and FINS (all *P* for trend < 0.0005). The characteristics of the participants with and without breast cancer are shown in **Supplementary Table 1**.

**Table 1:** Baseline characteristics of the study population according to the quartile of the TyG index.

	Q1	Q2	Q3	Q4	*P*_trend_
**TyG index**	8.00 ± 0.23	8.46 ± 0.10	8.82 ± 0.11	9.44 ± 0.39	<0.0001
**No. of participants**	35,546	35,544	35,548	35,546	
**Prevalence (%)**	0.39	0.61	0.56	0.72	<0.0001
**Age, years**	53.35 ± 9.28	56.15 ± 9.29	57.62 ± 9.10	58.38 ± 8.72	<0.0001
**BMI, kg/m^2^**	23.13 ± 3.26	24.06 ± 3.48	24.95 ± 3.61	25.79 ± 3.51	<0.0001
**Age at menarche, years**	15.36 ± 2.05	15.45 ± 2.07	15.45 ± 2.06	15.43 ± 2.08	<0.0001
**Current smokers, no. (%)**	423 (1.19)	448 (1.26)	500 (1.41)	579 (1.63)	<0.0001
**Current drinkers, no. (%)**	905 (2.55)	870 (2.45)	766 (2.15)	711 (2.00)	<0.0001
**Physical activity (moderate to vigorous), no. (%)**	4,407 (12.40)	4,257 (11.98)	4,174 (11.74)	4,008 (11.28)	0.0004
**Education status (high school or above), n (%)**	13,196 (37.12)	11,905 (33.49)	11,228 (31.59)	10,833 (30.48)	<0.0001
**Family history of breast cancer, n (%)**	230 (0.65)	242 (0.68)	211 (0.59)	222 (0.62)	0.52
**Healthy diet, no. (%)**	4,832 (16.58)	4,484 (15.42)	4,258 (14.52)	4,125 (13.88)	<0.0001
**FPG, mmol/L**	5.20 ± 0.54	5.44 ± 0.62	5.65 ± 0.79	6.33 ± 1.87	<0.0001
**2h-PG, mmol/L**	6.47 ± 1.78	7.09 ± 2.12	7.79 ± 2.57	9.49 ± 4.19	<0.0001
**HbA1c, %**	5.63 ± 0.44	5.75 ± 0.47	5.88 ± 0.57	6.26 ± 1.15	<0.0001
**TG, mmol/L**	0.75 (0.64–0.85)	1.10 (1.00–1.21)	1.52 (1.37–1.69)	2.39 (2.03–3.05)	<0.0001
**HDL-C, mmol/L**	1.49 ± 0.41	1.44 ± 0.35	1.36 ± 0.31	1.23 ± 0.27	<0.0001
**LDL-C, mmol/L**	2.51 ± 0.77	2.91 ± 0.80	3.12 ± 0.84	3.08 ± 0.92	<0.0001
**TC, mmol/L**	4.44 ± 1.06	4.93 ± 1.01	5.19 ± 1.02	5.47 ± 1.11	<0.0001
**FINS, µU/mL**	5.3 (4.0–7.0)	6.4 (4.8–8.5)	7.5 (5.6–10.0)	9.1 (6.8–12.3)	<0.0001
**Postmenopausal, no. (%)**	14,370 (50.73)	17,743 (65.19)	19,619 (73.05)	20,601 (77.76)	<0.0001
**Breastfeeding, no. (%)**	29,308 (87.76)	29,586 (88.42)	29,707 (88.51)	29,673 (88.65)	0.0016
**Number of childbirths, no. (%)**					<0.0001
**0–1**	8,154 (22.94)	7,389 (20.79)	6,806 (19.15)	6,492 (18.26)	
**2–4**	23,806 (66.97)	23,878 (67.18)	24,036 (67.62)	24,205 (68.09)	
**≥5**	3,586 (10.09)	4,277 (12.03)	4,706 (13.23)	4,849 (13.65)	

Data were presented as means (standard deviations) or medians (interquartile ranges) for continuous variables, or numbers (percentages) for categorical variables. TyG index, triglyceride glucose index; BMI, body mass index; FPG, fasting plasma glucose; 2h-PG, 2-h postload glucose; HbA1c, glycated hemoglobin; TG, triglyceride; HDL-C, high-density lipoprotein cholesterol; LDL-C, low-density lipoprotein cholesterol; TC, total cholesterol; FINS, fasting insulin.

Spearman correlation analysis was conducted to estimate the potential metabolic factors that influence the TyG index. It showed that the TyG index was positively correlated with 2h-PG, HbA1c, LDL-C and TC, FINS, and HOMA-IR (r ≤ 0.5) but negatively correlated with HDL-C (r = −0.3, **Table 2**).

**Table 2:** Spearman correlation analysis of potential metabolic factors influencing TyG.

	Coefficient	*P* value
**Age, years**	0.22	<0.0001
**BMI, kg/m^2^**	0.31	<0.0001
**Age at menarche, years**	0.013	<0.0001
**2h-PG, mmol/L**	0.39	<0.0001
**HbA1c, %**	0.32	<0.0001
**HDL-C, mmol/L**	-0.30	<0.0001
**LDL-C, mmol/L**	0.25	<0.0001
**TC, mmol/L**	0.35	<0.0001
**FINS, µU/mL**	0.43	<0.0001
**HOMA-IR**	0.50	<0.0001

BMI, body mass index; FPG, fasting plasma glucose; 2h-PG, 2-h postload glucose; HbA1c, glycated hemoglobin; TG, triglyceride; HDL-C, high-density lipoprotein cholesterol; LDL-C, low-density lipoprotein cholesterol; TC, total cholesterol; FINS, fasting insulin; homeostatic model assessment-insulin resistance (HOMA-IR).

**Figure 2:**
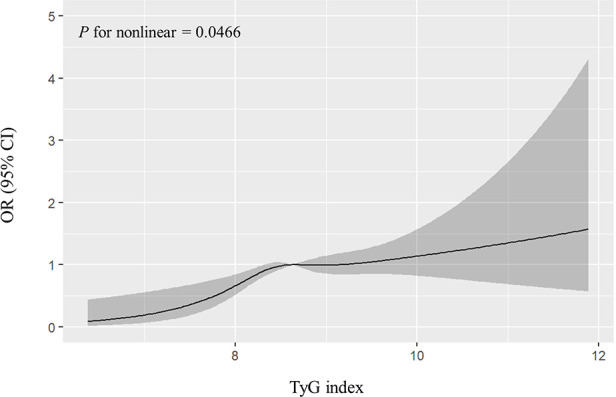
Non-linear dose–response relationship between the TyG index and breast cancer. The solid lines represent a fitted relationship, and the shadows represent the 95% confidence interval. TyG index, triglyceride glucose index.

The prevalence of breast cancer for women across quartile of the TyG index was 0.39%, 0.61%, 0.56%, and 0.72%, respectively. The association of the TyG index by quartile and the risk of breast cancer is given in **Table 3**. In unadjusted and age-adjusted models, both higher TyG index was associated with increased risk of breast cancer. After adjusting for potential covariates (multivariable-adjusted model 1), the ORs (95% CIs) of breast cancer in higher quartiles versus the lowest quartiles were 1.53 (1.20–1.95), 1.43 (1.11–1.83), and 1.73 (1.34–2.23), respectively. These associations persisted after further adjusting for reproductive factors; the ORs (95% CIs) of breast cancer in higher quartiles versus the lowest quartiles were 1.51 (1.14–2.00), 1.44 (1.08–1.93), and 1.61 (1.19–2.17), respectively. The results even remained significant after further adjusting for diabetes duration (**Supplementary Table 2**). The trends across quartiles were significant for the TyG index (*P* for trend < 0.0001). Each 1 SD increment of the TyG index was associated with a 29% increase in the risk of breast cancer in the fully adjusted model. The RCS analysis results showed that there was a non-linear relationship between the TyG index and breast cancer (*P* for non-linear < 0.05, **Figure 2**). In addition, when comparing the ORs of the TyG index, TG, HbA1c, and FPG, the ORs of higher levels of the TyG index stood out the most, indicating that the TyG index may have a superior discriminative ability for breast cancer (**Supplementary Table 3**). The PR (95% CI) of breast cancer associated with quartiles of the TyG index was similar (**Supplementary Table 4**). Per SD increasement of the TyG index was associated with a 16% increased prevalence of breast cancer (adjusted PR, 1.16; 95% CI, 1.05–1.28; **Supplementary Table 4**).

**Table 3:** Association between TyG index and risk of prevalent breast cancer.

	Q1	Q2	Q3	Q4	*P*_trend_	Per 1 SD increase
**TyG index (range)**	<8.28	8.28–8.63	8.64–9.02	>9.02		
**Case, n**	138	218	198	255		
**Prevalence (%)**	0.39	0.61	0.56	0.72	<0.0001	
**Unadjusted model**	1.00	1.58 (1.28–1.96)	1.44 (1.16–1.79)	1.85 (1.51–2.28)	<0.0001	1.38 (1.23–1.54)
**Age-adjusted model**	1.00	1.52 (1.22–1.88)	1.35 (1.08–1.68)	1.72 (1.39–2.12)	<0.0001	1.32 (1.18–1.49)
Multivariable-adjusted model 1^*^	1.00	1.53 (1.20–1.95)	1.43 (1.11–1.83)	1.73 (1.34–2.23)	<0.0001	1.34 (1.15–1.55)
Multivariable-adjusted model 2^**^	1.00	1.51 (1.14–2.00)	1.44 (1.08–1.93)	1.61 (1.19–2.17)	<0.0001	1.29 (1.08–1.53)

TyG index, triglyceride glucose index.

^*^Adjusted for age, BMI, smoking status (current smoker or not), drinking status (current drinker or not), physical activity (moderate to vigorous or none to mild), family history of breast cancer, healthy diet (yes or no), 2h-PG, HbA1c, and HDL-C.

^**^Adjusted for age, BMI, smoking status (current smoker or not), drinking status (current drinker or not), physical activity (moderate to vigorous or none to mild), family history of breast cancer, healthy diet (yes or no), 2h-PG, HbA1c and HDL-C, age at menarche, menopausal status (yes or no), and number of childbirths and breastfeeding (yes or no).

**Figure 3:**
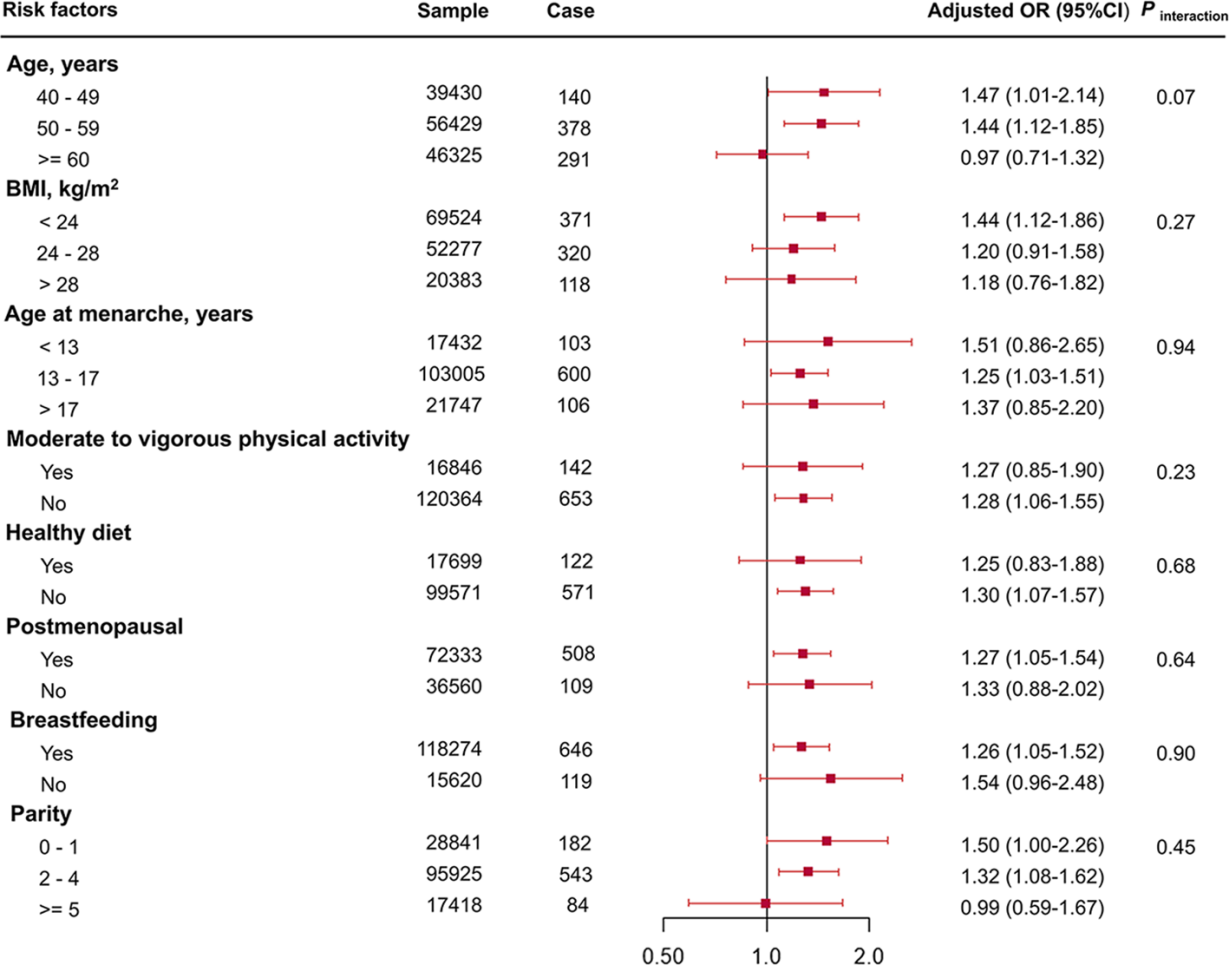
Association of TyG index per 1 SD increase with breast cancer risk stratified by potential risk factors. TyG index, triglyceride glucose index; OR, odds ratio; 95% CI, 95% confidence interval; BMI, body mass index.

In the stratification analysis (**Figure 3**), the association was stronger in women aged 50–59 years, with a BMI of lower than 24 kg/m^2^, age at menarche of 13–17 years old, those who were without moderate to vigorous physical activity and healthy diet, and those who were postmenopausal, breastfeeding, and have given birth to two to four children. There was no evidence of statistical interaction between risk factors and the TyG index (all *P* for interaction > 0.05). In addition, it seems that the association was significant among those without family history of breast cancer, rather than those with family history of breast cancer. However, no interaction between family history of breast cancer and TyG index was observed (*P* for interaction = 0.52, **Supplementary Table 5**).

In sensitivity analysis, the results were remained significantly when participants with IR, hyperglycemia, or dyslipidemia were excluded, when participants had both IR and hyperglycemia were excluded, and when participants had both IR and dyslipidemia were excluded. In individuals without IR, hyperglycemia, and dyslipidemia, each 1 SD increase in the TyG index is associated with a 1.36-fold increase in breast cancer risk (OR = 2.36; 95% CI 1.44–3.87), after adjustment for confounders (**Table 4**).

**Table 4:** Sensitivity analyses of association between every per-unit TyG index value increase and risk of prevalent breast cancer.

Sensitivity analyses	Total (n)	Cases (n)	Prevalence (%)	OR (95% CI)^**^
Excluding participants with IR (defined as HOMA-IR ≥2.5)	111,040	575	0.52	1.25 (1.003–1.55)
Excluding participants with hyperglycemia	82,626	397	0.48	1.40 (1.08–1.81)
Excluding participants with dyslipidemia	91,723	451	0.49	1.51 (1.07–2.13)
Excluding participants with IR and hyperglycemia	72,809	339	0.47	1.44 (1.08–1.93)
Excluding participants with IR and dyslipidemia	55,052	276	0.50	1.51 (1.07–2.13)
Excluding participants with IR, hyperglycemia and dyslipidemia	52,964	213	0.40	2.36 (1.44–3.87)

TyG index, triglyceride glucose index; IR, insulin resistance; HOMA-IR, homeostasis model assessment of insulin resistance.

^**^Adjusted for age, BMI, smoking status (current smoker or not), drinking status (current drinker or not), physical activity (moderate to vigorous or none to mild), family history of breast cancer, healthy diet (yes or no), 2h-PG, HbA1c and HDL-C, age at menarche, menopausal status (yes or no), and number of childbirths and breastfeeding (yes or no).

## 4 Discussion

In this study, we found that the TyG index was significantly associated with prevalent risk of breast cancer, even in those without discernible IR, hyperglycemia, and dyslipidemia. Our findings provide the first evidence of the association between the TyG index and breast cancer in a large Chinese middle-aged and elderly population.

Preclinical and clinical studies suggested complex associations between diabetes, especially type 2 diabetes with breast cancer (15). Our results showed that patients with breast cancer have significantly higher insulin levels than those without breast cancer, which is consistent with the previous evidence (7). As part of the important causes of diabetes, hyperinsulinemia and IR have been shown to be risk factors for breast cancer (4–7, 16). A study reported that compared with the lowest quartile, the highest quartile of insulin and HOMA-IR was associated with the greatest risk of breast cancer among 5,064 Chinese women (7). In addition, two studies from the Women’s Health Initiative (WHI) suggested that elevated levels of insulin and HOMA-IR may be a risk factor for postmenopausal breast cancer (5, 6), whereas glucose levels showed no association with this risk (6). A post genome-wide gene–environment interaction study among 11,109 postmenopausal women from the WHI identified single-nucleotide polymorphisms of HOMA-IR in combination with lifestyle as synergistic factors for breast cancer risk (4). However, a cohort study among 7,894 women from the Atherosclerosis Risk in Communities (ARIC) showed that there is no association between fasting insulin level and breast cancer incidence (17). This indicates that single indicators such as insulin or glucose may not be a good indicator for breast cancer in different populations, whereas the association between HOMA-IR and breast cancer suggests that it may be more beneficial to use multifactor indicators.

TyG index, a composite indicator based on TG and FPG, was reported to be an excellent surrogate indicator for IR that is economical and practical. Recently, a multicenter case–control study among 432 women from Indonesia showed that TyG index >8.87 was associated with risk of breast cancer (9). A cross-sectional Chinese study also found that the increasing TyG index was positively correlated with the heightening risk of breast cancer, but they used patients with breast disease rather than the general population (18). Furthermore, a hospital-based study among 510 Turkey patients with benign breast lesions or breast cancer indicated the predictive effect of the TyG index (cutoff was 8.628) in distinguishing benign and malignant lesions of the breast (19). However, in a large 17-year prospective study of 510,471 individuals from six European cohorts, the highest quintile of TyG index (>9.1) was not associated with the risk of postmenopausal breast cancer, compared with the lowest quintile (10). In our analysis, the prevalence of breast cancer in the highest quartile (>9.02) was nearly twice as high as in the lowest quartile (0.72% vs. 0.39%). Moreover, those in the highest quartile showed a significant association between TyG index and breast cancer, compared with the lowest quartile, and these risks remained mostly significant after adjustment for confounding factors. This result suggested that further examinations may be prioritized in women with a TyG index greater than 9.02. Our study firstly confirmed the association of the TyG index with breast cancer in a large Chinese general population more than 140,000 women, which provides powerful new evidence.

Reproductive risk factors, such as menstruation, number of births, and breastfeeding, have been previously reported to be associated with breast cancer risk (20). However, these conclusions remain controversial. The number of births can modulate breast cancer risk, and with some reduction with breastfeeding (21). In contrast, other studies have pointed out that the relationships between reproductive risk factors and breast cancer etiology are complex. Age at diagnosis of breast cancer appears to modify the effect of number of births; the elevated risk is not observed in women under 25 years of age (22). Moreover, the risk of breastfeeding on breast cancer varies among different populations (23, 24). In our analysis, when we further adjusted for age at menarche, menopausal status, number of childbirths, and breastfeeding, the association of the TyG index with breast cancer was attenuated but still significant. This suggested that the association between TyG index and breast cancer could not affected by these factors. In addition, we did not observe interactions between these risk factors and the TyG index for breast cancer risk. This finding is partially in line with several studies that found a null association of reproductive risk factors with breast cancer (9, 24). However, we found that the TyG index each 1 SD increase is associated with higher risk of breast cancer in postmenopausal women, in contrast to previous European reports that there is no association between TyG index and breast cancer (10). It could be partially due to the racial and population differences, which suggested that the association may be influenced by ethnic background. Additionally, higher risks were also observed in those with menarche at age 13–17, with breastfeeding, and had two to four children. This suggests that the TyG index might account at least partly for the additional risk despite these reproductive factors. Thus, TG or glucose level lowering appears as an additional target in women at high reproductive risk.

A previous study has reported that low physical activity is also a risk factor for breast cancer (20). Our findings supported this view because we found that the association of the TyG index with breast cancer was more predominant in women with low physical activity compared with women with moderate or vigorous physical activity, and the proportion of low physical activity was highest in the highest quartile of the TyG index. Due to the lack of relevant information, some other risk factors were not addressed in our study, including pathological type, genetic mutations, and exposure to steroid hormones (20). There is a long debate on whether oral contraceptives increase the risk of breast cancer; however, hormonal therapy for climacteric symptoms has been shown to increase the risk of breast cancer (25). A population-based study from Turkey confirmed the predictive effect of the TyG index in distinguishing benign and malignant lesions of the breast, which suggested that the TyG index may have greater predictive value for malignant breast lesions (19). The predictive role of the TyG index in distinguishing breast cancer gene mutation subtypes needs to be explored in future studies.

Specially, given that the cross-sectional study design could not confirm the causal relationship between TyG index and breast cancer, we further examined this association in those who were without IR, which is defined as HOMA-IR < 2.5 (26). We found that this association remained significant in women with HOMA-IR < 2.5. It is known that there are many risk factors for breast cancer in addition to IR. As a part of the TyG index, TG was also associated with breast cancer (27). Therefore, in the non-IR population defined by HOMA-IR, the risk of breast cancer may also be high. Our results suggested that the TyG index could be a potential marker for breast cancer risk among Chinese women and even perform better than HOMA-IR. Specially, the TyG index each 1 SD increase was significantly associated with a 2.36-fold greater risk of breast cancer among women without discernible IR, hyperglycemia, or dyslipidemia, after adjustment for confounders. It is worth noting that in addition to our study, there is still lack of evidence for this association in populations with a relatively healthy metabolic status. The reason for this association becoming stronger may be due to the fact that individuals with IR, hyperglycemia, and dyslipidemia are those who typically have more metabolic risk factors (28), which would obscure the association of the TyG index in the risk of breast cancer. Moreover, tumor cells required more energy compared with normal cells; several oncoproteins in breast cancer could promote the glycolytic process to provide substrates to highly proliferative cancer cells (29), which may lower glucose levels in patients with more advanced breast cancer. Importantly, this means that the TyG index, as a composite indicator, has the potential to serve as a warning marker for early prevention of breast cancer in relatively healthy women.

Mechanisms linking diabetes, especially type 2 diabetes, and breast cancer have been reported in previous studies (30, 31). These associations include the biological effects of type 2 diabetes on breast cancer risk and progression. At present, there are three potential mechanisms of association between type 2 diabetes and breast cancer, including the insulin pathway, insulin-like growth factor (IGF) pathway, and sex-hormone regulation (15). The influence of insulin on breast cancer is mainly based on the effect of IR or hyperinsulinemia, which activates the extracellular-related-kinase cascade and the AKT pathway through activation of the insulin receptor or the IGF receptor in breast cancer (15). The high risk of cancer in insulin-resistant individuals may also be due to overproduction of reactive oxygen species (ROS) (32). The increased production of mitochondrial ROS may affect the metastasis and recurrence pathway of breast cancer (33). In addition, overexpression of insulin receptor induces malignant transformation of mammary epithelial cell lines. Diabetes could also cause high plasma-free estrogen concentrations by increasing the production of sex hormones, which in turn activate the estrogen receptor (15). As part of the TyG index, TG also influences breast cancer through the AKT pathway via the G protein-coupled receptor (34). In addition, recent evidence has highlighted the importance of chronic inflammation in breast cancer pathobiology, such as NLRP3 inflammasome and cytokine oncostatin M, which are involved in breast cancer signaling and reprogramming the tumor microenvironment (35, 36).

The main strength of our study is the large sample size of more than 140,000 women from the 25-region community-based population, which represents the distribution of different regions in China. Nevertheless, there are some limitations in our study. Firstly, given the cross-sectional study design, we could not evaluate the causal relationship and directly compare the TyG index with HOMA-IR or other IR indices. However, we discerned an association between the TyG index and breast cancer in women without insulin resistance, as defined by HOMA-IR, and observed robust results. Further prospective studies are needed to identify the causal relationship between TyG index and breast cancer in larger Chinese population. Secondly, we were not able to categorize the pathological types of breast cancer. Thirdly, as our study was conducted in middle-aged and elderly women, we cannot generalize the results to younger women.

In conclusion, this is the first study that confirmed the association between the TyG index and the prevalent risk of breast cancer within a cohort exceeding 140,000 Chinese women. Moreover, our findings reveal that an elevated TyG index remains correlated with breast cancer among women without discernible IR, hyperglycemia, and dyslipidemia.

## Data availability statement

The original contributions presented in the study are included in the article/**Supplementary material**. Further inquiries can be directed to the corresponding authors.

## Ethics statement

The studies involving humans were approved by Institutional Review Board of the Ruijin Hospital, Shanghai Jiao Tong University School of Medicine. The studies were conducted in accordance with the local legislation and institutional requirements. The participants provided their written informed consent to participate in this study.

## Author contributions

JL: Writing – review & editing, Validation, Supervision, Funding acquisition. XW: Writing – original draft, Visualization, Formal analysis, Data curation, Conceptualization. SYW: Writing – original draft. LL: Writing – original draft. XJ: Writing – review & editing, Data curation. CH: Writing – review & editing, Data curation. HQ: Writing – review & editing, Data curation. HL: Writing – review & editing, Investigation, Data curation. RZ: Writing – review & editing, Investigation, Data curation. ML: Writing – review & editing, Investigation, Data curation. YX: Writing – review & editing, Investigation, Data curation. MX: Writing – review & editing, Investigation, Data curation. LLC: Writing – review & editing, Data curation. TZ: Writing – review & editing, Data curation. RH: Writing – review & editing, Data curation. ZY: Writing – review & editing, Data curation. LS: Writing – review & editing, Data curation. QS: Writing – review & editing, Data curation. XY: Writing – review & editing, Data curation. LY: Writing – review & editing, Data curation. TW: Writing – review & editing, Investigation, Data curation. ZZ: Writing – review & editing, Investigation, Data curation. JZ: Writing – review & editing, Investigation, Data curation. GQ: Writing – review & editing, Data curation. QW: Writing – review & editing, Data curation. GC: Writing – review & editing, Data curation. MD: Writing – review & editing, Investigation, Data curation. XT: Writing – review & editing, Data curation. ZG: Writing – review & editing, Data curation. FS: Writing – review & editing, Data curation. XG: Writing – review & editing, Data curation. ZL: Writing – review & editing, Data curation. YQ: Writing – review & editing, Data curation. LC: Writing – review & editing, Data curation. XH: Writing – review & editing, Data curation. YH: Writing – review & editing, Data curation. QL: Writing – review & editing, Data curation. GW: Writing – review & editing, Data curation. YZ: Writing – review & editing, Data curation. CL: Writing – review & editing, Data curation. YW: Writing – review & editing, Data curation. SLW: Writing – review & editing, Data curation. TY: Writing – review & editing, Data curation. HD: Writing – review & editing, Data curation. JJZ: Writing – review & editing, Data curation. YM: Writing – review & editing, Data curation. GN: Writing – review & editing, Validation, Project administration, Funding acquisition. WW: Writing – review & editing, Supervision, Project administration, Funding acquisition. YB: Writing – review & editing, Supervision, Funding acquisition. YC: Writing – review & editing, Supervision, Funding acquisition.
