# Supplementary material for: Association between triglyceride glucose index and breast cancer in 142,184 Chinese adults: findings from the REACTION study
Source: Front Endocrinol (Lausanne). 2024 Jun 6;15:1321622. doi: 10.3389/fendo.2024.1321622 (PMC11186986; doi:10.3389/fendo.2024.1321622)
Supplement: Supplementary file 1 [file Table_1.docx]

**Supplementary Table 1. Baseline characteristics of the study population with or without prevalent breast cancer.**

|  | **Breast cancer** | **No breast cancer** | ***P* value** |
| --- | --- | --- | --- |
| **No. of participants** | 809 | 141,375 |  |
| **Age, years** | 58.02±8.74 | 56.36±9.31 | <0.0001 |
| **BMI, kg/m^2^** | 24.61±3.35 | 24.48±3.61 | 0.28 |
| **Age at menarche, years** | 15.18±2.06 | 15.42±2.06 | 0.0013 |
| **Current smokers, no. (%)** | 7 (0.87) | 1,943 (1.37) | 0.21 |
| **Current drinkers, no. (%)** | 12 (1.48) | 3,240 (2.29) | 0.13 |
| **Physical activity (moderate to vigorous), no. (%)** | 142 (17.55) | 16,704 (11.82) | 0.0079 |
| **Education status (high school or above), n (%)** | 352 (43.51) | 46,810 (33.11) | 0.11 |
| **Family history of breast cancer, n (%)** | 18 (2.22) | 887 (0.63) | <0.0001 |
| **Healthy diet, no. (%)** | 122 (17.60) | 17,577 (15.08) | 0.064 |
| **FPG, mmol/L** | 5.72±1.16 | 5.65±1.17 | 0.078 |
| **2h-PG, mmol/L** | 8.26±3.33 | 7.71±3.04 | <0.0001 |
| **HbA1c, %** | 5.97±0.75 | 5.88±0.76 | 0.0011 |
| **TG, mmol/L** | 1.37 (1.01-2.06) | 1.28 (0.92-1.82) | <0.0001 |
| **HDL-C, mmol/L** | 1.35±0.36 | 1.38±0.35 | 0.018 |
| **LDL-C, mmol/L** | 2.92±0.89 | 2.90±0.87 | 0.54 |
| **TC, mmol/L** | 5.06±1.15 | 5.01±1.12 | 0.21 |
| **TyG index** | 8.72 (8.39-9.14) | 8.63 (8.28-9.02) | <0.0001 |
| **FINS,** **µU/mL** | 7.9 (5.7-10.8) | 6.9 (5.0-9.6) | <0.0001 |
| **Postmenopausal, no. (%)** | 508 (82.33) | 71,825 (66.34) | <0.0001 |
| **Breastfeeding, no. (%)** | 646 (84.44) | 117,628 (88.36) | 0.0008 |
| **Number of childbirths, no. (%)** |  |  | 0.039 |
| **0-1** | 182 (22.50) | 28,659 (20.27) |  |
| **2-4** | 543 (67.12) | 95,382 (67.47) |  |
| **≥5** | 84 (10.38) | 17,334 (12.26) |  |

BMI, body mass index; FPG, fasting plasma glucose; 2h-PG, 2-hour postload glucose; HbA1c, glycated hemoglobin; TG, triglyceride; HDL-C, high-density lipoprotein cholesterol; LDL-C, low-density lipoprotein cholesterol; TC, total cholesterol; TyG index, triglyceride glucose index; FINS, fasting insulin.

**Supplementary Table 2. Association between TyG index and risk of prevalent breast cancer after full adjustment plus diabetes duration.**

|  | **ORs** | **95% CIs** | ***P* value** |
| --- | --- | --- | --- |
| **Q1** | 1.00 | 1.00 | <0.0001 |
| **Q2** | 1.51 | 1.14-2.00 |  |
| **Q3** | 1.45 | 1.09-1.94 |  |
| **Q4** | 1.62 | 1.20-2.18 |  |
| **Per 1 SD increase** | 1.14 | 1.04-1.24 | <0.0001 |

Adjusted for age, BMI, smoking status (current smoker or not), drinking status (current drinker or not), physical activity (moderate to vigorous or none to mild), family history of breast cancer, healthy diet (yes or no), 2h-PG, HbA1c and HDL-C, age at menarche, menopausal status (yes or no), number of childbirths, breastfeeding (yes or no), and diabetes duration. TyG index, Triglyceride glucose index; ORs, odds ratios; 95% CIs, 95% confidence intervals.

**Supplementary Table 3. Association between metabolic index and risk of prevalent breast cancer.**

| **Risk factors** | **Q1** | **Q2** | **Q3** | **Q4** | ***P*_trend_** | **Per 1 SD increase** |
| --- | --- | --- | --- | --- | --- | --- |
| **TG** | 1.00 | 1.66 (1.34-2.06) | 1.45 (1.17-1.81) | 1.81 (1.47-2.24) | <0.0001 | 1.12 (1.07-1.18) |
| **HbA1c** | 1.00 | 1.12 (0.90-1.40) | 1.26 (1.04-1.52) | 1.53 (1.26-1.85) | <0.0001 | 1.13 (1.05-1.22) |
| **FPG** | 1.00 | 1.13 (0.92-1.38) | 1.23 (1.01-1.50) | 1.31 (1.07-1.60) | <0.0001 | 1.05 (1.00-1.10) |
| **TyG index** | 1.00 | 1.58 (1.28-1.96) | 1.44 (1.16-1.79) | 1.85 (1.51-2.28) | <0.0001 | 1.38 (1.23-1.54) |

The results were performed by using univariate logistic regression analysis.

TG, triglyceride; HbA1c, glycated hemoglobin; FPG, fasting plasma glucose; TyG index, triglyceride glucose index.

**Supplementary Table 4. Prevalence ratio of breast cancer associated with TyG index.**

|  | **Q1** | **Q2** | **Q3** | **Q4** | **Per 1 SD increase** |
| --- | --- | --- | --- | --- | --- |
| **Unadjusted model** | 1.00 | 1.58 (1.28-1.96) | 1.43 (1.16-1.79) | 1.85 (1.51-2.28) | 1.20 (1.13-1.28) |
| **Age-adjusted model** | 1.00 | 1.51 (1.22-1.88) | 1.35 (1.08-1.68) | 1.71 (1.39-2.12) | 1.18 (1.10-1.25) |
| **Multivariable-adjusted model 1^*^** | 1.00 | 1.53 (1.20-1.95) | 1.43 (1.11-1.83) | 1.72 (1.34-2.23) | 1.18 (1.08-1.29) |
| **Multivariable-adjusted model 2^**^** | 1.00 | 1.50 (1.14-2.00) | 1.44 (1.08-1.93) | 1.61 (1.19-2.17) | 1.16 (1.05-1.28) |

Results are presented as prevalence ratio (95% confidence interval).

TyG index, Triglyceride glucose index.

^*^Adjusted for age, BMI, smoking status (current smoker or not), drinking status (current drinker or not), physical activity (moderate to vigorous or none to mild), family history of breast cancer, healthy diet (yes or no), 2h-PG, HbA1c and HDL-C.

^**^Adjusted for age, BMI, smoking status (current smoker or not), drinking status (current drinker or not), physical activity (moderate to vigorous or none to mild), family history of breast cancer, healthy diet (yes or no), 2h-PG, HbA1c and HDL-C, age at menarche, menopausal status (yes or no), number of childbirths and breastfeeding (yes or no).

**Supplementary Table 5. Association between TyG index and risk of prevalent breast cancer stratified by family history of breast cancer.**

| **Family history of breast cancer** | **Sample** | **Case** | **Per 1 SD increase** | ***P* for interaction** |
| --- | --- | --- | --- | --- |
| **Yes** | 905 | 18 | 2.44 (0.67-8.93) | 0.52 |
| **No** | 141279 | 791 | 1.28 (1.07-1.52) |  |

Adjusted for age, BMI, smoking status (current smoker or not), drinking status (current drinker or not), physical activity (moderate to vigorous or none to mild), healthy diet (yes or no), 2h-PG, HbA1c and HDL-C, age at menarche, menopausal status (yes or no), number of childbirths, breastfeeding (yes or no), and diabetes duration. TyG index, Triglyceride glucose index.
